# A Gram-Scale Limonene Production Process with Engineered *Escherichia coli*

**DOI:** 10.3390/molecules25081881

**Published:** 2020-04-18

**Authors:** Jascha Rolf, Mattijs K. Julsing, Katrin Rosenthal, Stephan Lütz

**Affiliations:** 1 Chair for Bioprocess Engineering, Department of Biochemical and Chemical Engineering, TU Dortmund University, D-44227 Dortmund, Germany; jascha.rolf@tu-dortmund.de (J.R.); mattijs.julsing@wur.nl (M.K.J.); katrin.rosenthal@tu-dortmund.de (K.R.); 2 Wageningen Food & Biobased Research, Wageningen University & Research, 6708 WG Wageningen, The Netherlands

**Keywords:** monoterpenes, limonene, glycerol, mevalonate pathway, reaction engineering, bioprocess, biocatalyst, two-liquid phase fermentation, in situ product removal

## Abstract

Monoterpenes, such as the cyclic terpene limonene, are valuable and important natural products widely used in food, cosmetics, household chemicals, and pharmaceutical applications. The biotechnological production of limonene with microorganisms may complement traditional plant extraction methods. For this purpose, the bioprocess needs to be stable and ought to show high titers and space-time yields. In this study, a limonene production process was developed with metabolically engineered *Escherichia coli* at the bioreactor scale. Therefore, fed-batch fermentations in minimal medium and in the presence of a non-toxic organic phase were carried out with *E. coli* BL21 (DE3) pJBEI-6410 harboring the optimized genes for the mevalonate pathway and the limonene synthase from *Mentha spicata* on a single plasmid. The feasibility of glycerol as the sole carbon source for cell growth and limonene synthesis was examined, and it was applied in an optimized fermentation setup. Titers on a gram-scale of up to 7.3 g·L_org_^−1^ (corresponding to 3.6 g·L^−1^ in the aqueous production phase) were achieved with industrially viable space-time yields of 0.15 g·L^−1^·h^−1^. These are the highest monoterpene concentrations obtained with a microorganism to date, and these findings provide the basis for the development of an economic and industrially relevant bioprocess.

## 1. Introduction

Monoterpenes are volatile, lipophilic compounds in the essential oils of plants, which often find application as flavors and fragrances in food, cosmetics, and household chemicals. Limonene is the predominant monoterpene in the essential oils of citrus fruits and can be found in oaks, pines, and spearmint as well. Recently, limonene has been investigated as a promising alternative or additive for solvents [1] and jet fuels [2,3,4]. Limonene also shows antimicrobial properties [5], can be easily functionalized because of its two double bonds [6], and thus finds application as a building block for several commodity chemicals and pharmaceuticals. The oxygenated derivatives of limonene show potent pharmaceutical activities. As an example, perillyl alcohol, which can be obtained by the regiospecific oxygenation of limonene via whole-cell biotransformation [7,8], has proven anti-cancer properties [9]. The application of monoterpenes as starting materials for industrially or pharmaceutically relevant compounds requires efficient synthesis routes [10]. Nowadays, limonene is mainly produced as a by-product of orange juice production. However, the establishment of new applications will lead to a rapidly growing global market. The low concentrations of monoterpenes in natural sources make their isolation often economically unfeasible. Chemical synthesis might offer alternative production strategies. However, the chemical synthesis of these complex and often chiral molecules is typically difficult, involves many synthesis steps, and suffers from low yields. In order to ensure a stable and sustainable limonene supply, the development of a biotechnological process for limonene synthesis complements the traditional production route. Moreover, such a process could serve as a basis for the production of other monoterpenes of interest and subsequent selective functionalization.

**Figure 1:**
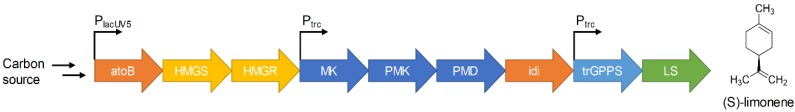
The heterologous mevalonate (MVA) pathway and limonene synthase introduced into *Escherichia coli* for the production of (S)-limonene. Acetoacetyl-CoA synthase from *E. coli* (atoB), HMG-CoA (hydroxymethylglutaryl-CoA) synthase from *Saccharomyces cerevisiae* (HMGS), an N-terminal truncated version of HMG-CoA reductase from *S. cerevisiae* (HMGR), mevalonate kinase (MK), phosphomevalonate kinase (PMK), phosphomevalonate decarboxylase from *S. cerevisiae* (PMD), isopentenyl diphosphate isomerase from *E. coli* (idi), a truncated and codon-optimized version of geranyl pyrophosphate synthase from *Abies grandis* (trGPPS), and a truncated and codon-optimized version of limonene synthase from *Mentha spicata* without the plastidial targeting sequence (LS).

During recent years, recombinant microbial strains have been engineered for limonene synthesis [11]. The production of isoprenoids with bacterial hosts was challenged by the low supply of the common precursors isopentenyl pyrophosphate (IPP) and dimethylallyl pyrophosphate (DMAPP) via the native 2-C-methyl-d-erythritol 4-phosphate (MEP) pathway. Higher precursor availability was realized by the introduction of a heterologous mevalonate (MVA) pathway from *Saccharomyces cerevisiae* in *Escherichia coli*, and isoprenoid titers above 100 mg·L^−1^ were achieved for the first time. A nine-enzyme pathway was constructed on three plasmids to produce amorpha-4,11-diene, which is the sesquiterpene precursor to artemisinin, an antimalarial drug [12]. Based on this study, an equivalent set of plasmids was designed to produce limonene with recombinant *E. coli* [8]. The pathway was optimized by balancing the involved enzymes in several iterative steps, and the number of plasmids was reduced to a single plasmid (Figure 1). Cultivations of the engineered *E. coli* strain in shake flasks using glucose as carbon source in a complex medium resulted in limonene titers of up to 400 mg·L^−1^.

Willrodt et al. constructed another *E. coli* strain harboring a two-plasmid system (pBAD:LS, pET24:AGPPS2) and operated a two-liquid phase fed-batch setup with a minimal medium in a stirred-tank bioreactor [13]. In this study, the addition of an inert organic phase was used to prevent product inhibition, toxicity effects, and the evaporative loss of limonene. Diisononyl phthalate (DINP) was selected as a biocompatible organic carrier solvent because of its favorable partition coefficient and lack of detectable impact on the growth of *E. coli* [14]. Final limonene concentrations of 1350 mg·L^−1^ were reached with glycerol as the sole carbon source, which was an almost 4-fold increase in limonene formation compared to that from glucose fermentations using the same strain. The use of glycerol resulted in a prolonged growth and production phase, leading to a more stable process with a maximum space-time yield of about 40 mg·L^−1^·h^−1^ for carbon-limited cultivation [13].

Rational strain optimization, as well as reaction engineering, demonstrated the potential of the biotechnological production of monoterpenes. Nevertheless, space-time yields and product titers are still not applicable for industrial production. Additionally, data obtained at the bioreactor scale are rare. This study aims at the development of a feasible bioreactor scale process for monoterpene production with a recombinant *E. coli* strain that is genetically optimized for limonene synthesis.

## 2. Results

### 2.1. Influence of Inducer Concentration on Limonene Yields

**Figure 2:**
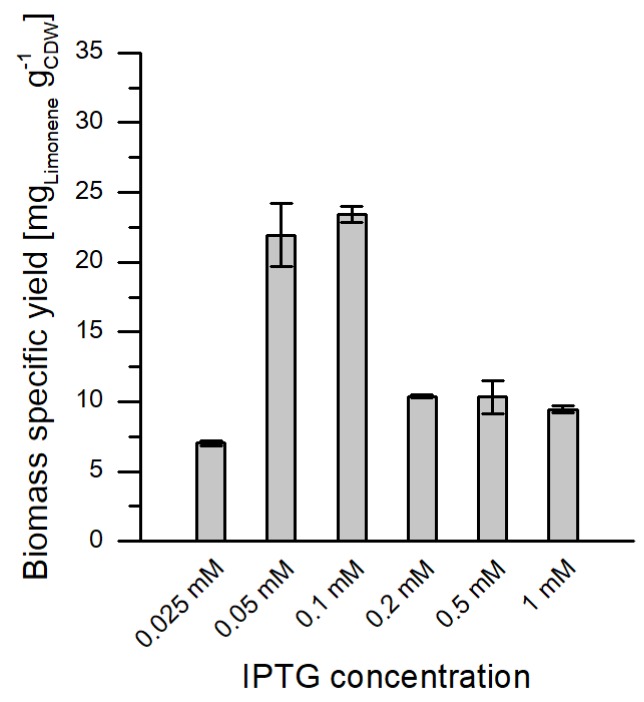
Biomass specific yields for different concentrations of the inducer isopropyl β-d−1-thiogalactopyranoside (IPTG) after 12 h of cultivation. Two-liquid phase shake flask fermentations with *E. coli* BL21 (DE3) pJBEI-6410 in M9 minimal medium with 0.5% *w*/*v* glucose as the sole carbon source. The error bars relate to biological duplicates.

Previous studies with a single plasmid strain (*E. coli* DH1 pJBEI-6409) cultivated in complex medium elucidated that low inducer concentrations (0.025 mM isopropyl β-d−1-thiogalactopyranoside (IPTG)) resulted in the highest limonene titers [8]. It was hypothesized that the amount of LacI produced by the single copy of *lacI* in the vector might not be enough to fully repress all three promoters in pJBEI-6409. The fully expressed MVA and limonene pathway at high IPTG levels could be too stressful for efficient limonene production. In the present study, different IPTG levels (0.025, 0.05, 0.1, 0.2, 0.5, and 1 mM) were tested for the optimal expression of heterologous genes. In comparison to the mentioned study, a different single plasmid strain was used (*E. coli* BL21 (DE3) pJBEI-6410), which carries a version of pJBEI-6409 harboring ampicillin resistance instead of chloramphenicol resistance. Furthermore, fermentations were carried out in M9 minimal medium instead of a complex medium. It turned out that the highest biomass specific yields could be obtained with IPTG concentrations of 0.05 mM and 0.1 mM (Figure 2). These values are high compared to the reported inducer concentrations for the producer strain *E. coli* DH1 pJBEI-6409 [8]. Following the hypothesis of Alonso-Gutierrez et al., the higher optimal inducer levels could be explained by a higher *lacI* expression level [8]. In contrast to *E. coli* DH1, the host strain *E. coli* BL21 (DE3) carries a Lac regulatory construct in its genome [15]. This operon includes *lacI*^q^, which is a mutant of *lacI* with a 10-fold higher expression level that leads to a lower basal expression of T7 RNA and therefore to a more tightly controlled expression [16]. For the following experiments, the inducer concentration of 0.1 mM IPTG was chosen to ensure sufficient induction during bioreactor experiments.

### 2.2. Glycerol as the Sole Carbon Source for Fermentative Limonene Production

**Figure 3:**
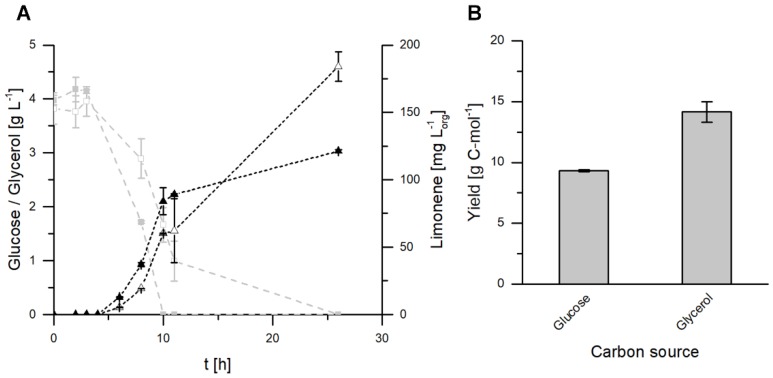
Two-liquid phase shake flask fermentations with *E. coli* BL21 (DE3) pJBEI-6410 in M9 minimal medium with either 0.5% *w*/*v* glucose (closed symbols) or glycerol (open symbols) as the sole carbon source. (**A**) Limonene concentrations (▲, △) in the organic phase and carbon source (■, □) concentrations were determined at regular intervals. (**B**) Carbon specific limonene yields after 26 h of cultivation. The error bars relate to biological duplicates.

**Figure A1:**
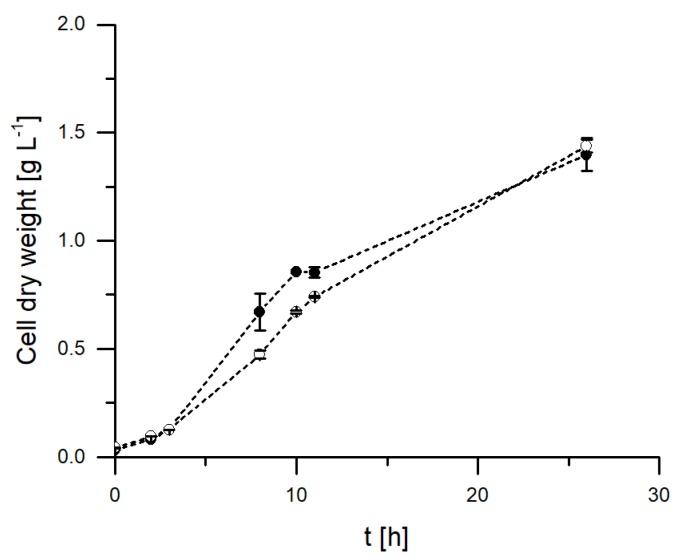
Two-liquid phase shake flask fermentations with *E. coli* BL21 (DE3) pJBEI-6410 in M9 minimal medium with either 0.5% *w*/*v* glucose (closed symbols, ●) or glycerol (open symbols, ○) as the sole carbon source. Cell dry weights were determined at regular intervals. The error bars relate to biological duplicates.

A prolonged growth phase of *E. coli* and higher product concentrations with glycerol as the sole carbon source were described by Willrodt et al. in an aforementioned study [13]. In order to investigate if these influences can also be observed with *E. coli* BL21 (DE3) pJBEI-6410, shake flask experiments were carried out using either glucose or glycerol as the sole carbon source. In the case of glucose, the substrate was consumed completely after 10 h, whereas glycerol was still present in the fermentation medium after 11 h. Finally, the carbon source was fully consumed in both cultivations. The growth curves were also similar (Appendix, Figure A1). Using glucose for the carbon supply resulted in a final limonene concentration of 121 ± 1 mg·L_org_^−1^ in the organic phase (Figure 3A). By comparison, the fermentation with glycerol showed a prolonged production phase, resulting in a final limonene concentration of 184 ± 11 mg·L_org_^−1^. The limonene yields relative to the carbon source were 9.3 ± 0.1 g·C-mol^−1^ and 14.2 ± 0.8 g·C-mol^−1^, for glucose and glycerol, respectively (Figure 3B). These results confirm previous observations that glycerol is the better choice as a carbon source for fermentative limonene production with *E. coli*.

### 2.3. Fermentative Limonene Production in a Stirred-tank Reactor

**Figure 4:**
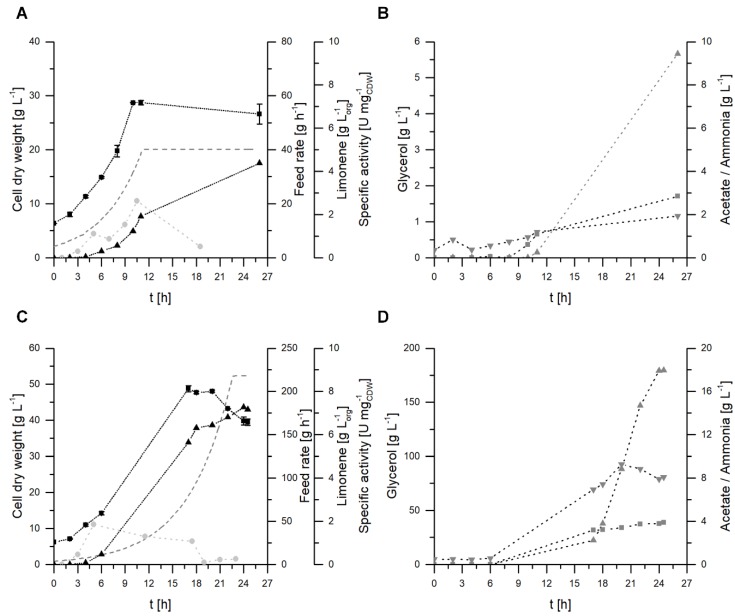
Two-liquid phase fed-batch fermentation with *E. coli* BL21 (DE3) pJBEI-6410 in M9 minimal medium. Cell dry weight (CDW) (■), limonene concentrations (▲) in the organic phase, glycerol (▲), acetate (▼), and ammonium (■) concentrations were determined at regular intervals. The specific activities (●) were calculated for distinct time points throughout the fermentation time. The feed rate is displayed as well (dotted line). (**A)** and (**B)** display the initial fed-batch fermentation (D = 0.18 h^−1^), whereas (**C**) and (**D**) display the optimized fed-batch fermentation with a lower feed rate (D = 0.15 h^−1^) and additional trace element supply. The error bars for CDW relate to two independent measurements.

The first attempt to produce limonene with *E. coli* BL21 (DE3) pJBEI-6410 and glycerol as the sole carbon source in a two-liquid phase fed-batch setup was carried out in a 3.1 L reactor with 1 L of M9 minimal medium and 0.5 L of the organic carrier solvent diisononyl phthalate (DINP). A carbon limited exponential feed was applied with a calculated growth rate of 0.18 h^−1^ (Figure 4A,B), which was based on a substrate specific biomass production rate obtained from an initial batch cultivation. Heterologous gene expression was induced with the addition of 0.1 mM IPTG after two hours of fed-batch cultivation. After 10 h of exponential growth, no further increase in biomass was observed. The growth rate for this period was 0.15 h^−1^ and a final cell dry weight (CDW) of 28.7 g·L^−1^ was achieved. No accumulation of glycerol was detected until this time point. Acetate formation was suppressed during growth. After 11 h, growth stopped, and the exponential feed was set constant. During the next 15 h, glycerol accumulated, followed by acetate formation, leading to final concentrations of 5.7 g·L^−1^ glycerol and 2.8 g·L^−1^ acetate. The ammonium concentration increased during the fermentation from 0.4 to 1.9 g·L^−1^, probably due to the pH regulation with ammonia as a result of acetate and carbon dioxide formation. The specific activity of limonene synthesis increased after induction with IPTG and reached a maximum of 2.6 U g_CDW_^−1^ by the end of exponential growth. However, significant limonene formation was still observed for the non-growing cells, and a final limonene concentration of 4.4 g·L_org_^−1^ was achieved. Concentrations of limonene were determined for the organic phase volume, because of the significant dilution of the aqueous phase due to the addition of the feed solution. The cell growth and the production of limonene seemed to be limited by a yet unidentified mechanism, which might be the accumulation of limonene or another compound related to the biosynthesis of limonene—such as an intermediate or metabolite—or a limitation caused by the depletion of a medium component.

To test whether intracellular limonene, terpene intermediates, or the expression of heterologous pathway genes influence cell growth, a glycerol-limited fed-batch fermentation was carried out without the induction of the heterologous pathway (data not shown). Although heterologous gene expression was not induced, 222 mg·L_org_^−1^ limonene was produced during the exponential growth. These small amounts are probably caused by leaky expression of the pathway genes. Nearly identical CDW was achieved, while 20-fold less limonene was produced. Therefore, toxification by intermediates of the MVA pathway or intracellular limonene can be excluded.

In order to overcome the growth limitation, the parameters for the fermentation were changed to permit prolonged exponential growth, coupled with higher limonene concentrations. A carbon limited exponential feed was set up with a calculated growth rate of 0.15 h^−1^, which is lower than in the previous experiments (0.18 h^−1^) (Figure 4C,D). Furthermore, additional US* trace elements were supplied. After 6 and 16 h of cultivation, 2 and 1 mL of US* trace element solution were spiked, respectively. The exponential growth could be maintained for nearly 17 h, with a growth rate of 0.12 h^−1^. Thus, the addition of trace elements prolonged the growth phase, and a CDW of 48.8 g·L^−1^ was reached, whereas the second trace element spike did not affect the cells in their stationary phase. The specific activity quickly increased reaching a maximum of 1.8 U g_CDW_^−1^ after 5 h, before it decreased with ongoing fermentation. In contrast to in previous experiments, the specific activity could be maintained above 1 U g_CDW_^−1^ for a time period of more than 17 h. The prolonged production phase and higher biomass concentration resulted in 7.3 g·L_org_^−1^ limonene after 24 h by the end of the fermentation. To our knowledge, this is the highest limonene titer reported so far.

## 3. Discussion

### 3.1. Glycerol is a Suitable Carbon Source for Heterologous Limonene Production in Escherichia coli

The beneficial effect of glycerol as a sole or supplementary carbon source has been reported before for the fermentative production of carotenoids in MEP engineered *E. coli* strains [17,18,19], for sesquiterpenes [12] and for limonene [13] in MVA-engineered *E. coli* strains. The use of glycerol as a carbon source resulted in higher limonene formation rates, a prolonged growth phase, and increased stability compared to the same whole-cell biocatalyst growing on glucose [13].

In the present study, we were able to transfer this knowledge to a bioprocess with an optimized one plasmid strain and showed that, compared to glucose, glycerol is definitely the preferred carbon source for the production of limonene with *E. coli*. The transferability to the bioreactor scale was validated, and reaction engineering was performed to further increase the limonene titer. This led to the highest monoterpene concentration obtained with a microorganism to date. Next to availability and low cost, various advantages make glycerol an attractive carbon source for fermentation processes compared to glucose. Firstly, beneficial effects on the viability of cells and productivity of recombinant proteins were observed [20]. Secondly, glycerol does not show any catabolic repression in combination with lactose, which might be the preferred inducer for heterologous gene expression instead of IPTG due to a reduced stress level for the production host. Catabolite repression occurs when excess glucose is present and leads to reduced lactose uptake rates, which causes the decreased expression of recombinant proteins [21]. Finally, glycerol is a suitable carbon source for anaerobic fermentation with *E. coli* strains producing biofuels and highly reduced compounds. The high degree of reduction of carbon atoms in glycerol (κ = 4.67) provides a distinct advantage over glucose (κ = 4.00) in the absence of other electron acceptors [22]. *E. coli* strains are able to utilize glycerol in such conditions for cell growth and need a suitable sink for the excess reducing equivalents generated during the formation of biomass [23]. Therefore, the ability to form a highly reduced product is essential for the microorganism. Limonene has a high degree of reduction (κ = 5.60), so it would be a suitable product and sink for reducing equivalents in anaerobic glycerol fermentation. The anaerobic environment could have another beneficial effect regarding the toxicity of limonene. Whereas limonene itself has relatively little toxicity towards *E. coli* cells, the common oxidation product limonene hydroperoxide, which forms spontaneously in aerobic environments, shows highly antimicrobial effects [24]. In this study, the inhibitory effects of limonene hydroperoxide were not observed, due to efficient product extraction in the organic phase.

### 3.2. Progess to an Economic Limonene Production Process

The optimized bioreactor process described in this study resulted in limonene productivity exceeding the threshold for developing a profitable production process for fine chemicals (100 mg·L^-1^·h^−1^) [25] for the first time (Table 1). However, a techno-economic assessment stated that a biotechnological production process for limonene needs to have a space-time yield above 700 mg·L^−1^·h^−1^ and a 45% carbon specific yield to be competitive with established processes [26]. While our process already shows more than a fifth of the space-time yield required, the conversion of the carbon source into the product is still low, at less than 1%.

Different approaches to improve the yield have already been described. Non-growing but metabolically active *E. coli* cells can boost the production of the desired product due to reduced energy and carbon loss to biomass formation [28]. A fourfold increase in specific limonene yields relative to biomass was accomplished with this strategy. Moreover, further pathway debottlenecking and the optimization of the involved enzymes could increase the economics of the process. For example, the exchange of the geranyl pyrophosphate synthase with a neryl pyrophosphate synthase from *Solanum lycopersicum* led to increased limonene production with *E. coli* [27]. Another strategy to increase the product titer and the specificity of monoterpenes in *E. coli* was described by Chacón et al. [29]. The monoterpene geraniol was converted to the monoterpenoid geranyl acetate with an in vivo esterification and extracted in situ to an organic phase. Toxicity issues and the synthesis of by-products could be circumvented, resulting in a monoterpenoid concentration of 4.8 g·L^−1^. Similar approaches to the coupled synthesis and functionalization of limonene are described, which produce the valuable monoterpenoid perillyl alcohol [8,30].

The highest yields with more than 95% and titers of 15 g·L^−1^ were achieved with a cell-free system consisting of 27 purified enzymes, which convert glucose into monoterpenes [31]. Other systems which incorporate acetic acid as a starting building block for the cell-free synthesis of terpenes are described as well [32]. However, a major drawback is the need for purified enzymes, which are associated with additional costs and the further input of glucose needed to produce them. The need for enzyme purification can be avoided with the use of enzyme-enriched *E. coli* lysates, but this approach appeared to suffer from low product titers of 90 mg·L^−1^ [33]. Moreover, the involved enzymes are considered to have low stabilities in the in vitro environment, and cofactor regeneration could be a limiting aspect in cell-free applications as well [32]. The expensive cofactors CoA and NADPH must be effectively recycled in such systems, while the use of whole cells circumvents these drawbacks as the cofactors are regenerated by the primary metabolism. Therefore, microorganisms are preferred as the biocatalyst for the larger biotechnological production of limonene. Microbial hosts other than *E. coli* were recently investigated as producer strains, such as the cyanobacterium *Synechocystis* sp. [34] or the oleaginous yeast *Yarrowia lipolytica* [35], which was able to produce limonene from waste cooking oil. However, product titers were orders of magnitude lower compared to the processes based on engineered *E. coli*.

Next to the selection and optimization of the production system, a feasible bioprocess with high limonene titers involves the integrated development of in situ product removal strategies. Due to the high volatility and inhibitory effects on cell growth, the capturing of limonene during fermentation is required. Various methods are available, with two-liquid phase and gas stripping systems being especially suitable at higher scales [36]. In particular, two-liquid phase systems have the advantage that the products are effectively removed from the fermentation broth [37]. The choice of capturing method is also dependent on the further application of the product. If limonene is subsequently used as a pure compound, solvent-free systems might be the better choice, whereas application as an additive for, e.g., solvents might allow the use of the same solvent for in situ extraction [38]. In the present study, the in situ product removal strategy in combination with an engineered *E. coli* strain and a glycerol-limited fed-batch fermentation enabled the synthesis of the highest limonene concentration reported to date. Steps towards an economic process were made, and the potential of integrating already generated knowledge with the biotechnological production of terpenes was demonstrated.

## 4. Materials and Methods

### 4.1. Chemicals and Bacterial Strains

All chemicals used in this work were purchased from Carl Roth GmbH & Co. KG (Karlsruhe, Germany) and Merck KGaA (Darmstadt, Germany).

*E. coli* BL21 (DE3) harboring the plasmid pJBEI-6410 was used for all experiments. pJBEI-6410 carries the genes for the MVA pathway, a geranyl pyrophosphate synthase, and the limonene synthase from *Mentha spicata* [8]. It was a gift from Taek Soon Lee (RRID:Addgene_47049; http://n2t.net/addgene:47049).

### 4.2. Cultivation and Fermentative Limonene Production in Shake Flasks

For the cultivation in shake flasks, precultures were grown in 5 mL of LB medium (10 g·L^−1^ tryptone, 5 g·L^−1^ yeast extract, and 5 g·L^−1^ NaCl) with 100 µg·mL^−1^ ampicillin for 4 to 6 h at 37 °C and shaking at 200 rpm. LB precultures were transferred 1:500 to 250 mL baffled Erlenmeyer flasks with 50 mL M9 minimal medium (8.5 g·L^−1^ Na_2_HPO_4_ · 2H_2_O, 3 g·L^−1^ KH_2_PO_4_, 0.5 g·L^−1^ NaCl, 1 g·L^−1^ NH_4_Cl, 2 mL of 1 M MgSO_4_, and 1 mL of L^−1^ US* trace element solution) with either 0.5% *w*/*v* glucose or 0.5% *w*/*v* glycerol as the sole carbon source. The cultures were incubated overnight at 30 °C with shaking at 200 rpm. The M9 precultures were used to inoculate 80 mL of M9 minimal medium with the 0.5% *w*/*v* carbon source in 500 mL baffled shake flasks to an optical density of 0.11 at 600 nm (OD_600_). The main cultivations were performed at 30 °C with shaking at 200 rpm. Heterologous gene expression was induced by adding 0.1 mM isopropyl β-d−1-thiogalactopyranoside (IPTG) once an OD_600_ of 0.4–0.6 was reached. After induction, the cultures were overlaid with 20 mL of diisononyl phthalate (DINP), and incubation was continued. For sampling, the phases were separated by centrifugation (2 min, 4 °C, 11,000 × *g*). Prior to gas chromatography (GC) analysis, the organic phase was diluted in diethyl ether with 0.2 mM dodecane and dried over anhydrous Na_2_SO_4_. The aqueous phase was used for HPLC analysis. The cell dry weight was determined by measurement of the optical density at a wavelength of 600 nm (Libra S11 Visible Spectrophotometer, Biochrom, Cambridge, UK). One OD_600_ unit corresponded to 0.312 g_CDW_ L^−1^, with a linear range between 0.1 and 1.

### 4.3. Fermentative Limonene Production in a Stirred-Tank Reactor

Two-liquid phase fermentations at the bioreactor scale were carried out in a 3.1 L stirred-tank reactor (KLF 2000, Bioengineering AG, Wald, Switzerland). The tank reactor was equipped with two Rushton turbine stirrers. An M9 preculture was used to inoculate an initial batch cultivation, which was performed in 1 L of M9 minimal medium containing a 1.5% *w*/*v* carbon source at a starting OD_600_ of 0.11. The pH was set to 7.2 and controlled by the automatic addition of either 30% *v*/*v* phosphoric acid or 25% *v*/*v* ammonia hydroxide solution. Batch cultures were incubated at 30 °C, with shaking at 1800 rpm and an aeration rate of 1 vvm, until the batch phase was finished after 13 to 17 h (indicated by a steep pO_2_ increase). Before the start of the carbon-limited fed-batch fermentation, 1 mL of US* trace element solution was added. To keep the exponential growth rates between 0.18 and 0.2 h^−1^, a carbon-limited and controlled exponential feed with a 73% *w*/*v* carbon source and 19.6 g·L^−1^ MgSO_4_ · 7 H_2_O was started. The pO_2_ value was kept above 30% by adjusting the stirrer speed and the aeration rate. If desired, recombinant gene expression was induced by adding 0.1 mM IPTG after 2 h of exponential feeding, and 0.5 L of DINP were added subsequently. Antifoam A was added only in cases of excessive foaming. Regular sampling was carried out as described above.

### 4.4. Quantification of Limonene

The quantification of limonene in DINP was carried out with GC using a TRACE GC Ultra (Thermo Fisher Scientific Inc., Waltham, MA, USA), equipped with a FactorFour-5ms column (30 m, 0.25 mm, 0.25 μm, Varian, Inc., Palo Alto, CA, USA) and a flame ionization detector. Nitrogen was used as a carrier gas and the injection volume was set to 1 μL (80 °C, 5 min; 80–140 °C, 7.5 °C·min^−1^; 140–300 °C, 40 °C·min^−1^; 300 °C, 5 min). Samples of the organic phase were prepared in diethyl ether containing 0.2 mM dodecane as an internal standard. The quantification of limonene was performed using a standard curve of the ratio between commercial (S)-limonene (Merck KGaA, Darmstadt, Germany) and the internal standard.

### 4.5. Quantification of Glucose, Glycerol, Acetate, and Ammonia

The quantifications of glucose, glycerol, and acetate concentrations were performed by high performance liquid chromatography (HPLC) with a LaChrome Elite HPLC system (VWR, Darmstadt, Germany), equipped with a Trentec 308R-Gel.H column (Trentec Analysetechnik, Gerlingen, Germany) and a refractive index detector. The mobile phase consisted of 5 mM sulphuric acid. An isocratic method was used, with a flow rate of 1 mL min^−1^ and an injection volume of 20 μL. The column oven was set to 40 °C. Eluted components were quantified using standard curves for glucose, glycerol, and acetate.

Ammonia concentrations were determined by the method of Berthelot [39].

## Figures and Tables

**Table 1:** *Escherichia coli*-based fermentation setups for the production of limonene.

Strain	Plasmid System	Medium	Setup	c_Limonene_ [mg·L^−1^]	STY [mg·L^−1^ h^−1^]	Reference
*E. coli* DH1	pJBEI-6409	EZ-Rich / glucose	Batch—Shake flask	435	6	[8]
*E. coli* BL21 (DE3)	pBAD:LS, pET24:AGPPS2	M9 / glycerol	Fed-batch—STR	1350	23	[13]
*E. coli* BW25113 (DE3)	pMAP6, pISP6, pNLSt1	YM9 / glucose	Fed-batch—Shake flask	1290	15	[27]
*E. coli* BL21 (DE3)	pJBEI-6410	M9 / glycerol	Fed-batch—STR	3630	151	This study

STY: space-time yield; STR: stirred tank reactor.
